# Tcf7L2 is essential for neurogenesis in the developing mouse neocortex

**DOI:** 10.1186/s13064-018-0107-8

**Published:** 2018-05-11

**Authors:** Olga Chodelkova, Jan Masek, Vladimir Korinek, Zbynek Kozmik, Ondrej Machon

**Affiliations:** 1 Institute of Molecular Genetics of the Czech Academy of Sciences, Vídeňská 1084, 14200 Prague, Czech Republic; 2 Institute of Experimental Medicine of the Czech Academy of Sciences, Vídeňská 1084, 14200 Prague, Czech Republic; 3 Present address: Department of Biosciences and Nutrition, Karolinska Institutet, SE-14183 Huddinge, Sweden; 4 Laboratory of Eye Biology, Division BIOCEV, Institute of Molecular Genetics of the Czech Academy of Sciences, Prumyslova 595, Vestec, Czech Republic

**Keywords:** Neurogenenesis, Neocortex, Wnt signalling, Tcf7L1, Tcf7L2

## Abstract

Generation of neurons in the embryonic neocortex is a balanced process of proliferation and differentiation of neuronal progenitor cells. Canonical Wnt signalling is crucial for expansion of radial glial cells in the ventricular zone and for differentiation of intermediate progenitors in the subventricular zone. We detected abundant expression of two transcrtiption factors mediating canonical Wnt signalling, *Tcf7L1* and *Tcf7L2*, in the ventricular zone of the embryonic neocortex. Conditional knock-out analysis showed that Tcf7L2, but not Tcf7L1, is the principal Wnt mediator important for maintenance of progenitor cell identity in the ventricular zone. In the absence of Tcf7L2, the Wnt activity is reduced, ventricular zone markers Pax6 and Sox2 are downregulated and the neuroepithelial structure is severed due to the loss of apical adherens junctions. This results in decreased proliferation of radial glial cells, the reduced number of intermediate progenitors in the subventricular zone and hypoplastic forebrain. Our data show that canonical Wnt signalling, which is essential for determining the neuroepithelial character of the neocortical ventricular zone, is mediated by Tcf7L2.

**Electronic supplementary material:**

The online version of this article (10.1186/s13064-018-0107-8) contains supplementary material, which is available to authorized users.

## Introduction

The neocortex in mouse is formed during mid-embryogenesis in the prosencephalon as a multi-layer structure from cortical progenitors cells [1]. Neural progenitor cells in the cortical ventricular zone (VZ) termed radial glial cells (RGC) generate neurons directly during asymmetric mode of cell division giving rise to one progenitor daughter cell and one daughter neuron, or one intermediate progenitor cell (IPC). IPCs may undergo few rounds of cell division in the subventricular zone (SVZ) before terminal differentiation into postmitotic neurons [2–4]. In the early forebrain at embryonic stages (E) 8-11 days post coitum, neural progenitors divide symmetrically to rapidly expand the progenitor pool before the asymmetric mode of cell division is initiated [5]. Several signalling pathways including Wnt are involved in regulation of neurogenesis. At E8-11, canonical Wnt/β-catenin signalling promotes symmetric division in the forebrain [6–8] while later (from E12.5 onwards) it promotes terminal differentiation of IPC into neurons [9–12]. Transition of radial glial cells to IPCs is accompanied with the loss of adherens junctions and neuroepithelial identity [3]. Canonical Wnt signaling is mediated through activation of β-catenin and its translocation to the nucleus in which β-catenin associates with transcription factors of Tcf/Lef family and regulates expression of Wnt target genes. Classical loss-of-function studies of canonical Wnt signalling employ conditional deletion of β-catenin in various tissues including the neocortex [8, 13]. Since β-catenin is involved both in forming adherens junctions and in controlling transcription of Wnt target genes this approach is not suitable for addressing β-catenin role during neocortical development. Abrogation of canonical Wnt signalling downstream of β-catenin, however, may provide an experimental approach that does not perturb cell adhesion properties of radial glial cells. We therefore focused on the role of Tcf/Lef transcription factors in the neocortex. Although all four proteins bind to β-catenin and to identical DNA consensus binding site, Lef1 and Tcf7 (formerly named Tcf1) operate as transcriptional activators, Tcf7L1 (formerly Tcf3) acts as a repressor while Tcf7L2 (formerly Tcf4) may exhibit both functions depending on the cellular context [14, 15]. Lef1 is expressed in the tip of the medial cortical wall and it is crucial for the dentate gyrus development while the lateral cortex is normal in *Lef1*-/- mutants [16]. *Tcf7L1* and *Tcf7L2* are abundantly expressed in the forebrain while *Tcf7* is not [17, 18]. Here we examined the expression patterns of *Tcf7L1* and *Tcf7L2* in the neocortex at critical neurogenic stages between E13 and E17 and analyzed neural differentiation upon conditional inactivation of *TcF7L1*, *Tcf7L2* or both. We provide evidence that *Tcf7L2* as a transcriptional activator controls the neuroepithelial character of RGC progenitor zone and RGC proliferation.

## Results

### Tcf7L1 and Tcf7L2 are expressed in the VZ of the lateral and medial neocortical wall during embryonic neurogenesis

**Fig. 1:**
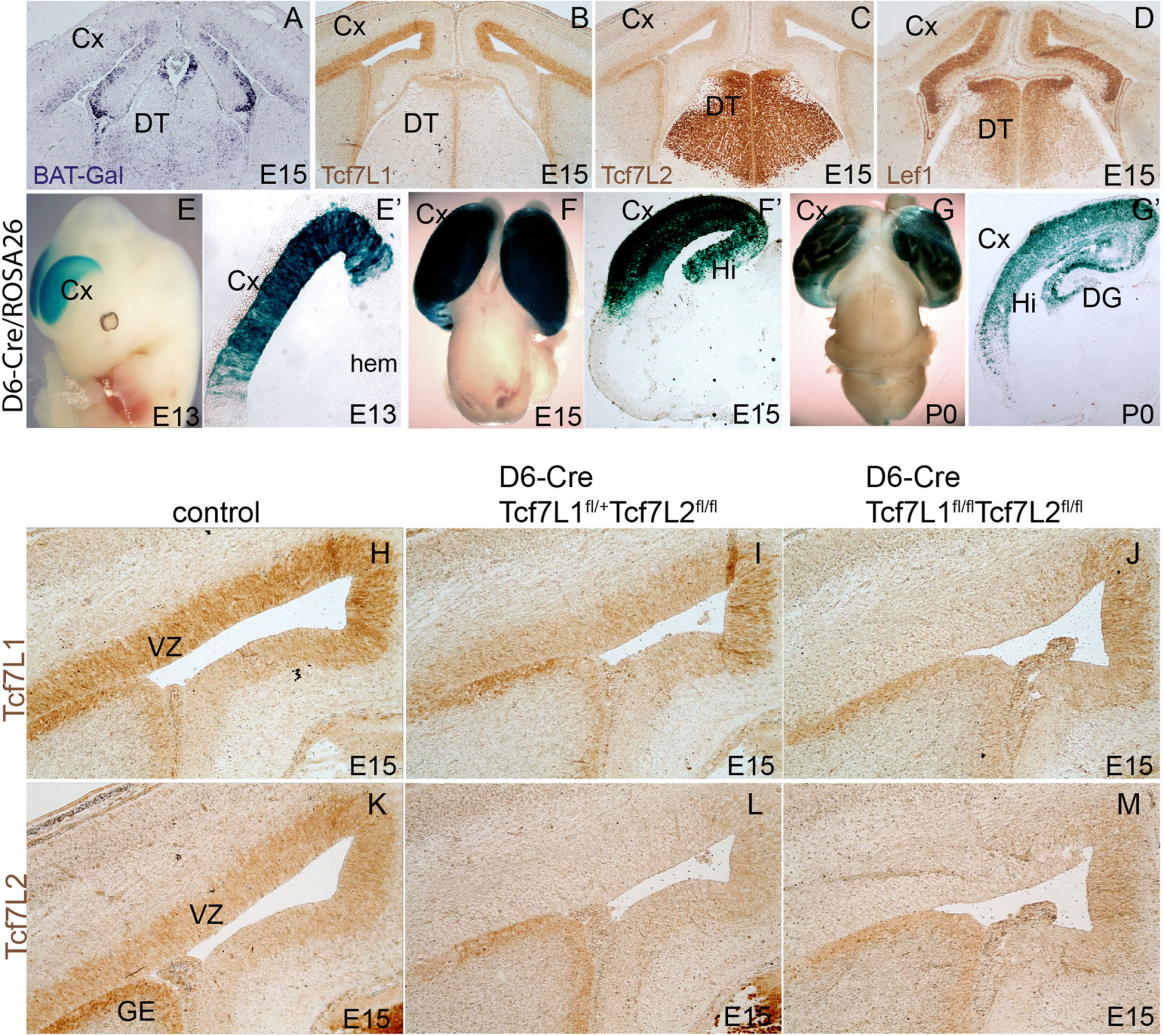
Tcf7L1 and TCF7L2 expression in the cortical VZ. **a** in situ hybridization on coronal cortical sections from BAT-Gal mice using *β*-*galactosidase* probe shows medial-to-lateral gradient of Wnt signalling. **b** Immunohistochemistry of Tcf7L1 showing strong expression in the VZ at E15. **c** Immunohistoechistry of Tcf7L2 showing expression in the VZ and strong expression in the dorsal thalamus (DT). **d** Lef1 immunohistochemistry at E15 shows high expression in the medial cortical wall that is reciprocal to that of Tcf7L1 and Tcf7L2. **e-g‘** Lineage tracing of D6-Cre/ROSA26 descendant cells in the neocortex at E13, E15 and newborns (P0) showing the Cre activity in the neocortex (Cx), hippocampus (Hi) and very weakly in the dentate gyrus (DG). **h-m** Immunohistochemistry with Tcf7L1 and Tcf7L2 antibodies at E15. Tcf7L1 is deleted in Tcf7L1^fl/fl^/Tcf7L2^fl/fl^ double mutants but not in Tcf7L1^fl/+^/Tcf7L2^fl/fl^ mutants while Tcf7L2 is deleted in both mutants documenting a high specificity of applied antibodies

Canonical Wnt activity in the mouse embryonic neocortex was mapped using BAT-Gal reporter mouse in which multiplicated Tcf consensus binding sites coupled to a minimal promoter drive the expression of the *β-galactosidase* gene [19]. The strongest activity was observed in the hem and the medial cortical wall that gradually declined laterally [9, 20]. This reflects a well known signalling centre in the hem expressing a number of Wnt proteins including Wnt3a [21]. We performed in situ hybridization on sections of the neocortex from BAT-Gal mice using antisense probe for *β-galactosidase* mRNA. Figure 1a illustrates the medial-high to lateral-low gradient of the Wnt activity in the mouse neocortex at E15. Next, we asked what Tcf/Lef factor may mediate canonical Wnt signalling in this brain region. Immunohistochemistry on cortical sections using specific antibodies revealed that *Lef1* expression correlates with medial-lateral gradient of BAT-Gal reporter (Fig. 1d). This is in the line with the idea that the hippocampus and the dentate gyrus are patterned by Wnt3a signalling through Lef1-regulated gene expression [16]. Tcf7L1 was strongly expressed in the lateral neocortical VZ and the signal declined in the medial wall and the hem (Fig. 1b, h). Tcf7L2 was abundant in the dorsal thalamus (DT) (Fig. 1c) corresponding to strong expression of *Tcf7L2* mRNA as detected by in situ hybridization [18]. Interestingly, we detected a clear presence of Tcf7L2 protein in the VZ of the neocortex and ganglionic eminences (GE) by immunohistochemistry on sections which was not previously indicated by in situ hybridization (Fig. 1c, k). Both Tcf7L1 and Tcf7L2 proteins were present in the VZ at analyzed stages from E13 to newborn stage P0 (data not shown).

### Conditional inactivation of TcfTL2 but not Tcf7L1 results in severe defects in the embryonic neocortex and hippocampus

The expression of *Tcf7L1* and *Tcf7L2* suggested that these two factors might control the transcriptional output of canonical Wnt signalling in the neocortex. To test this assumption we employed tissue-specific deletion of these *Tcf* factors. We crossed forebrain-specific Cre driver mouse line D6-Cre allowing loxP-recombination in the neocortex from E11.5 [22] with conditional alleles of *Tcf7L1* [23] and *Tcf7L2*. D6-Cre/ROSA26 lineage tracing in whole-mounts and on coronal sections of brains at E13.5, E15.5 and newborns (P0) illustrated Cre activity in the lateral and medial neocortex (Cx) and in the hippocampus (Hi) (Fig. 1e-g). Immunohistochemical analysis of the resulting D6-Cre/Tcf7L1^fl/+^/Tcf7L2^fl/fl^ crosses showed moderately reduced levels of Tcf7L1 expression while Tcf7L2 was not detectable. In D6-Cre/Tcf7L1^fl/fl^/Tcf7L2^fl/fl^ double mutants, however, both proteins were absent in the VZ in the area of D6-Cre recombination confirming efficient inactivation of *Tcf7L1* and *Tcf7L2* in the neocortex at E15 (Fig. 1h-m).

**Fig. 2:**
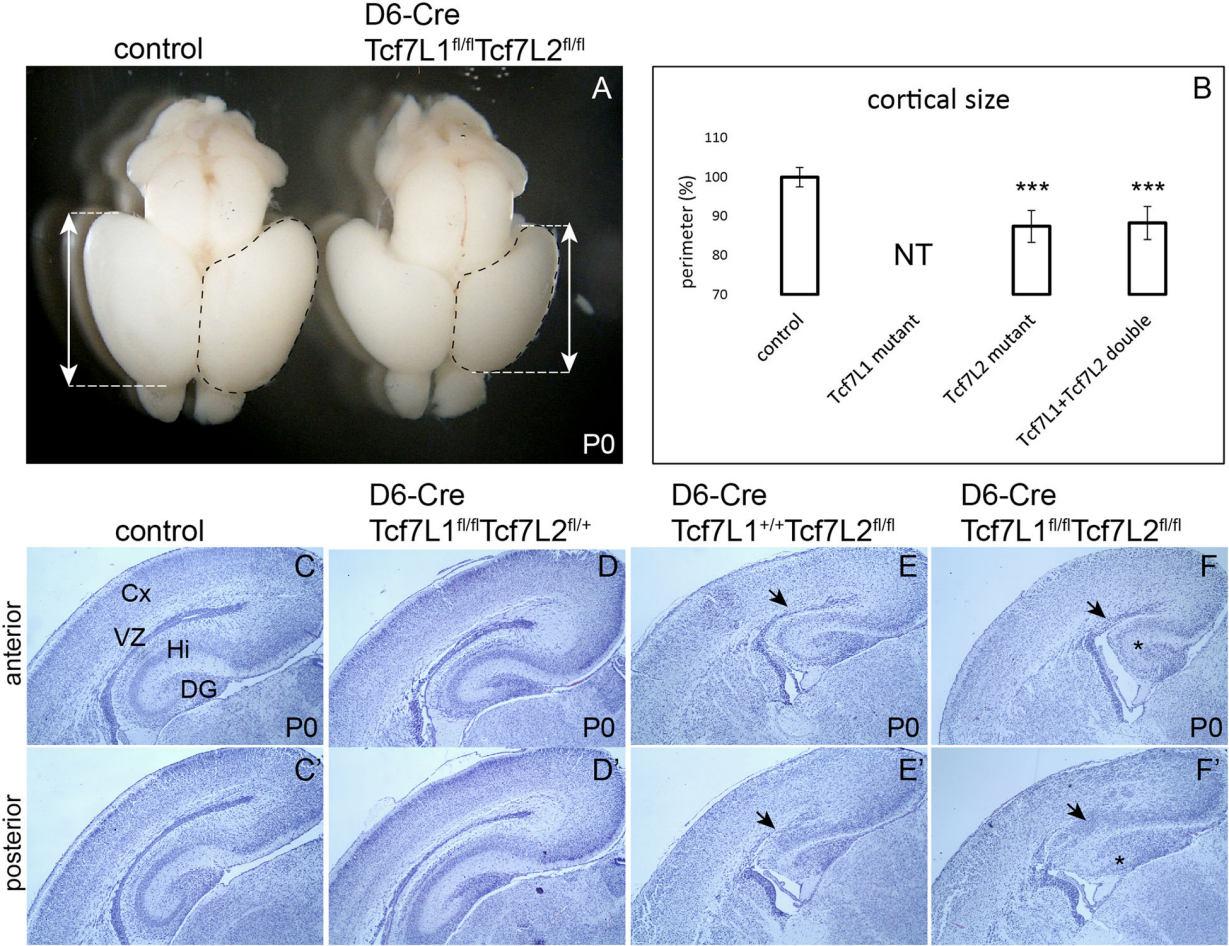
Hypoplasia of the neocortex and disruption of the ventricular zone in D6-Cre/Tcf7L1^fl/fl^/Tcf7L2^fl/fl^ mutants. **a** Smaller cortical lobes in Tcf7L1/Tcf7L2 double mutants dissected from newborn mice (P0). Arrows illustrate the length of cortices. The perimeter of cortices is labeled with a dashed line. **b** Statistical analysis of cortical size (perimeter) in Tcf7L2 single and Tcf7L1/Tcf7L2 double mutants with standard deviations. Student’s paired t-test: ***p < 0.001 compared to controls; controls *n* = 8 and either mutants *n* = 5. The size of Tcf7L1 cortices was not tested (NT). **c-f‘** Hematoxylin-eosin staining of coronal sections at P0 from controls (**c**-**c‘**), Tcf7L1 single mutants (**d**-**d‘**), Tcf7L2 single mutants (**e**-**e‘**) and Tcf7L1/Tcf7L2 double mutants (**f**-**f‘**). Arrows point at the disrupted VZ and asterisks show the impaired hippocampus

The phenotypic changes upon deletion of both Tcf factors were thoroughly examined at newborn stage P0. The brain size in D6-Cre/Tcf7L1^fl/fl^/Tcf7L2^fl/fl^ double mutants was smaller compared to control littermates as illustrated by the length of cortices (arrows) (Fig. 2a). Measurement of the perimeter of cortices in controls and mutants has confirmed statistically significant size reduction in single Tcf7L2 (87.4 +/- 4%) and double Tcf7L1/Tcf7L2 mutants (88.3 +/- 4.2%) compared to controls (Fig. 2b). On the other hand, the size difference between Tcf7L2 single and Tcf7L1/Tcf7L2 double mutants was not statistically significant (*p*-value: *p* = 0.75). Hematoxylin-eosin-stained coronal sections revealed severe changes in the brain morphology (Fig. 2c-f‘). Neocortical VZ and SVZ almost disappeared in double mutants (arrows in Fig. 2f-f‘). The neocortex was thinner and cortical layering was not distinct. The hippocampus was progressively reduced towards the posterior part in which it was almost absent (asterisk in Fig. 2f‘). This probably reflects the already reported higher recombination rate of D6-Cre driver in the posterior forebrain [22]. We further analyzed single mutants *Tcf7L1* (D6-Cre/Tcf7L1^fl/fl^/Tcf7L2^fl/+^) and single mutants *Tcf7L2* (D6-Cre/Tcf7L1^+/+^/Tcf7L2^fl/fl^). The phenotype in single *Tcf7L2* mutants resembled the double mutant (Fig. 2e-e‘, arrows). In contrast, the morphology in single *Tcf7L1* mutants appeared normal (Fig. 2d-d‘). These data suggest that Tcf7L2 is the principal transcription factor mediating canonical Wnt signaling in the neocortex.

### Neuroepithelium in the neocortical VZ is disintegrated in D6-Cre/Tcf7L2^fl/fl^ mutants and RGC proliferation is reduced

**Fig. 3:**
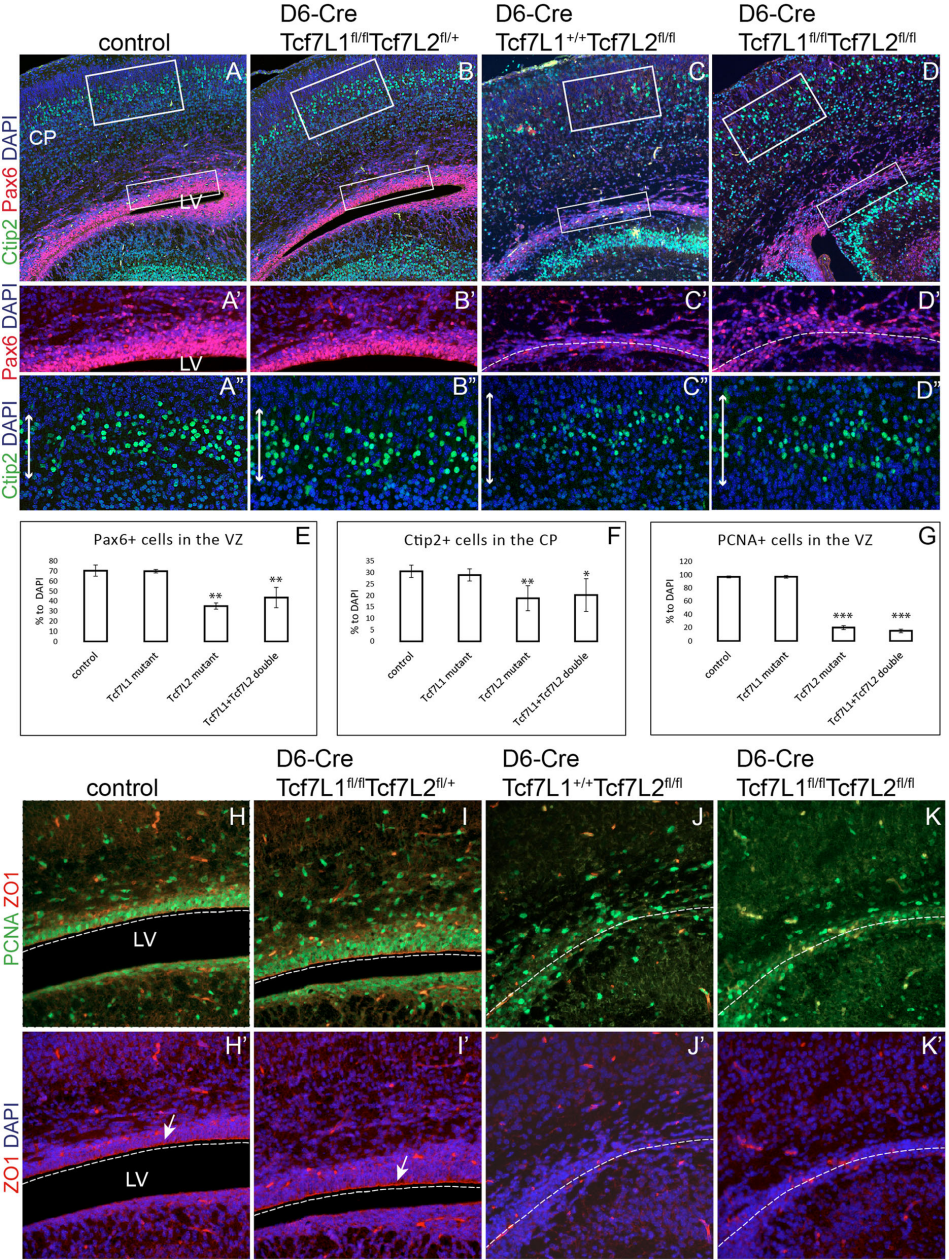
The neuroepithelial structure is severely impaired in the absence of Tcf7L2. **a-d‘** Pax6^+^ RGC and Ctip2^+^ cortical neurons are reduced upon inactivation of Tcf7L2 alone (**c**-**c**“) or upon simultaneous deletion of Tcf7L1 and Tcf7L2 (**d**-**d“**). Inactivation of Tcf7L1 alone had a little influence on RGC and neuronal populations (**b**-**b**“). Panels in **a‘** and **a“** display magnified framed rectangles in **a**. **e-g** Quantifications of Pax6^+^, Ctip2^+^ and PCNA^+^ dividing cells showing an average from three independent experiments with standard deviations. Student’s paired t-test: *p < 0.05, **p < 0.01, ***p < 0.001 compared to controls, *n* = 8. **h-k“ **Double immunofluorescent labelling of PCNA^+^ dividing cells and ZO1^+^ apical adherens junctions in RGC. Dashed lines represent potential ventricular lining. Vertical arrows depict the thickness of the cortical layers II-IV containing Ctip2^+^ neurons

To confirm the laminar patterning defect and the VZ disruption in the double mutants, we performed immunohistochemistry on coronal sections of brain at P0 using specific cellular markers. The number and position of Pax6-positive (Pax6^+^) RGC in the VZ were unaltered in single Tcf7L1 mutants (D6-Cre/Tcf7L1^fl/fl^/Tcf7L2^fl/+^) (Fig. 3b-b‘) compared to control littermates in which around 70% of all DAPI^+^ cells were Pax6+ (Fig. 3a-a‘, e). In contrast, the number of Pax6^+^ RGC was reduced to one half (35 +/- 3.1% of all cells) in single *Tcf7L2* mutants and to 43 +/- 10.1% in double mutants *Tcf7L1/Tcf7L2* (Fig. 3c-d‘, e). Statistical analysis of Pax6^+^ cell quantification confirmed no significant differences between controls and Tcf7L1 mutants (Student’s paired t-test: *p* value (p) = 0.81 and between Tcf7L2 and Tcf7L1/Tcf7L2 (*p* = 0.15) Fig. 3e). Next, we monitored the number and position of neurons in the cortical layers 5–3 labelled with Ctip2. Again, the number of Ctip2^+^ neurons remained unchanged in single *Tcf7L1* mutants (29 +/- 2.6%) compared to controls (30.6 +/- 2.7%, *p* = 0.45). On the other hand, single *Tcf7L2* and double *Tcf7L1/Tcf7L2* mutants displayed significant reduction of neurons to 18.8 +/- 5.4% and 20.2 +/- 7.1%, respectively, in the cortical plate (Fig. 3a”, b”, c”, d”). Quantifications of Ctip2^+^ cells are summarized in (Fig. 3f). Again, we observed no statistical significance in the number of Ctip2^+^ cortical neurons between Tcf7L2 and Tcf7L1/Tcf7L2 double mutants (*p* = 0.74). First phenotypic changes including decreased numbers of Pax6^+^ RGC and Ctip2^+^ neurons were observed at E17.5 (Additional file 1: Figure S1a-h“). At E15.5, however, we did not notice any dramatical alterations in the structure and cell composition of the neocortex of D6-Cre/Tcf7L1^fl/+^/Tcf7L2^fl/fl^ and D6-Cre/Tcf7L1^fl/fl^/Tcf7L2^fl/fl^ mutants as analyzed by immunofluorescence of Pax6^+^ RGCs, Ctip2^+^ or Tuj1^+^ cortical plate, Tbr2^+^ IPCs in the SVZ, PH3^+^ proliferating cells and ZO1^+^ adherens junctions (Additional file 2: Figure S2a-i‘).

Neuroepithelial progenitors in the VZ are characterized by adherens junctions at the apical side. These cell-cell junctions are maintained by polarized localization of cadherins and catenins and they can be labelled with, for instance, the ZO1 marker. We checked the integrity of adherens junctions by ZO1 immunofluorescence in the mutants at P0 stage. As depicted in Fig. 3h‘, i‘, j‘, k‘, ZO1 labelling at the apical side was completely lost in the lateral cortical wall in single *Tcf7L2* and double *Tcf7L1/Tcf7L2* mutants compared to controls. Again, single *Tcf7L1* mutants displayed normal ZO1-labelled lining of the apical side of RGC (arrow in Fig. 3i‘). Next, we calculated the number of dividing cells in the VZ using immunofluorescence with PCNA antibody. In controls and single *Tcf7L1* mutants, around 97% of RGC expressed PCNA marker and proliferated (Fig. 3h, i) whereas in single *Tcf7L2* and double *Tcf7L1/Tcf7L2* mutants, the number of dividing cells in the VZ was reduced to 20.1 +/- 2.9% and 15.1 +/- 2.6%, respectively (Fig. 3j, k). The summary of quantifications from three independent experiments is shown in Fig. 3g. In conclusion, *Tcf7L2* deletion caused severe reduction of RGC proliferation that was accompanied with disruption of the neuroepithelial structure of the VZ and neocortical stratification defects.

### The loss of Tcf7L2 in the embryonic neocortex leads to down-regulation of canonical Wnt signalling and reduction of radial glial cells in the VZ and intermediate progenitors in the SVZ

**Fig. 4:**
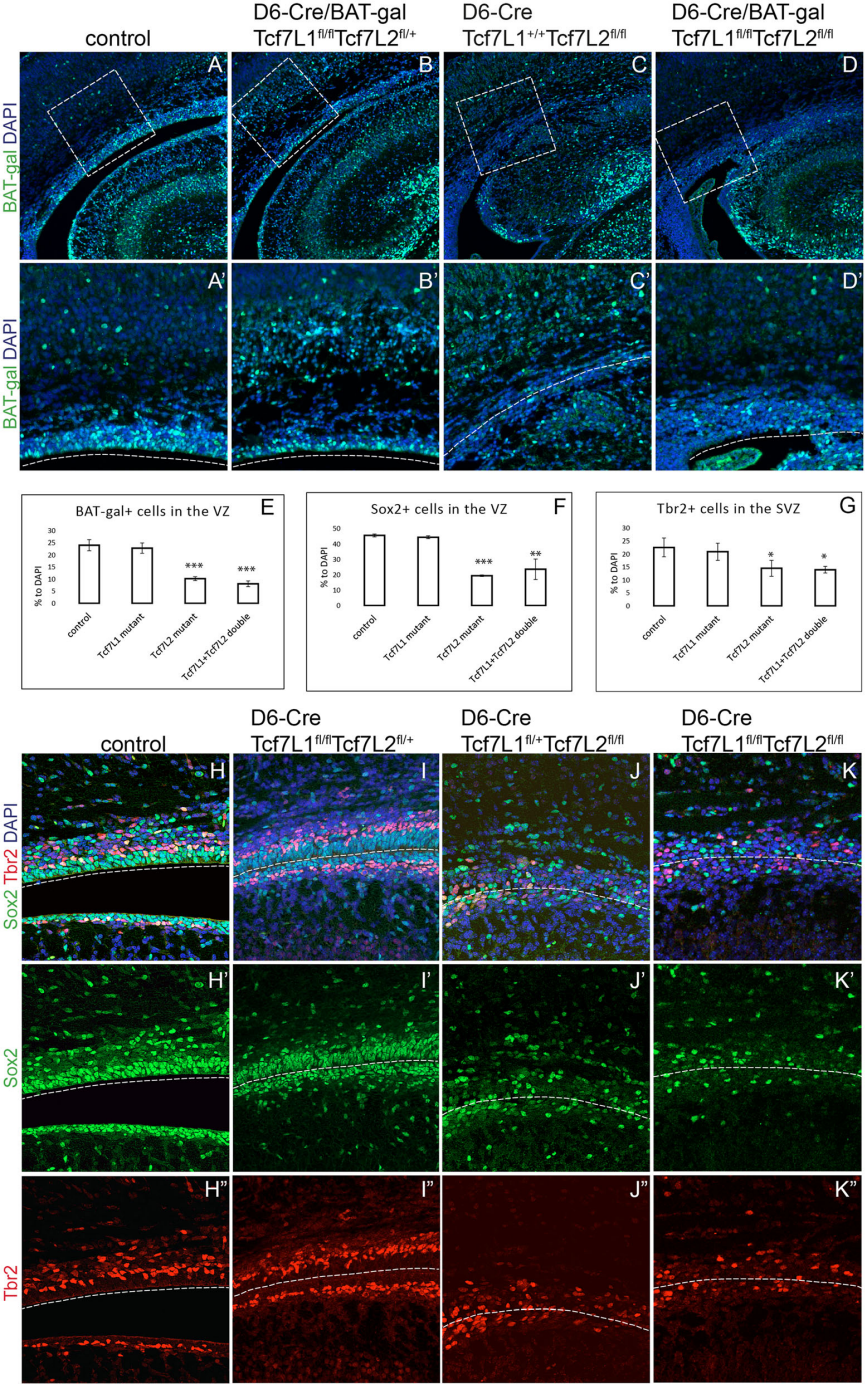
The loss of Tcf7L2 in the embryonic cortex leads to downregualtion of canonical Wnt signalling and reduction of radial glial cells and intermediate progenitors. **a-d‘** β–galactosidase immunofluorescence in the cortical VZ in BAT-Gal Wnt reporter mice is reduced after inactivation of Tcf7L2 alone (**c**-**c‘**) or upon simultaneous deletion of Tcf7L1 and Tcf7L2 (**d**-**d‘**). Inactivation of Tcf7L1 alone had a little effect on BAT-Gal Wnt reporter (**b**-**b**“). Panels in **a‘** display magnified framed rectangles in **a**. **e-g** Quantifications of BAT-Gal^+^, Sox2^+^ RGCs and Tbr2^+^ IPCs showing an average from three independent experiments with standard deviations. Student’s paired t-test: *p < 0.05, **p < 0.01, ***p < 0.001 compared to controls, *n* = 6. *P*-values between Tcf7L2 and Tcf7L1/Tcf7L2 mutants are: BAT-Gal^+^ *p* = 0.06, Sox2^+^ *p* = 0.34, Tbr2^+^ *p* = 0.80 showing no statistical significance. **h-k“ **Double immunofluorescent labelling of Sox2^+^ RGC and Tbr2^+^ IPCs. Dashed lines represent potential ventricular lining

Gain-of-function studies using constitutively active form of β-catenin demonstrated that proliferation of early neural progenitors is regulated by Wnt signalling [6, 24]. We therefore tested the Wnt activity upon the loss of *Tcf7L1* and *Tcf7L2*. For monitoring in vivo, we crossed the Wnt reporter mouse line BAT-Gal [19] to the D6-Cre/Tcf7L1^fl/fl^/Tcf7L2^fl/fl^ compound strain. The expression output of Wnt signalling was measured on brain sections at P0 using immunofluorescence with an antibody against β-galactosidase. In controls, BAT-Gal^+^ cells were localized to the VZ in which they represented around 24.1 +/- 2.2% of all DAPI^+^ cells (Fig. 4a-a‘) and the expression gradient corresponded to in situ hybridization shown in Fig. 1a. As expected, inactivation of *Tcf7L1* alone had negligible, if any, effect on the Wnt activity in the neocortical VZ with 22.9 +/- 2.1% of BAT-Gal^+^ cells (*p* = 0.55 compared to controls) (Fig. 4b-b‘). However, deletion of *Tcf7L2* or both *Tcf7L1/Tcf7L2* resulted in decrease to 10.2 +/- 0.7% and to 8.1 +/- 1.2% of all DAPI^+^ cells, respectively, of BAT-Gal^+^ cells (Fig. 4c-d‘). Quantifications from three independent experiments are summarized in Fig. 4e.

Proliferation and differentiation of IPC is dependent on Wnt signalling as well [9, 20]. We therefore immunotainstained intermediate progenitors using Tbr2 antibody in *Tcf7L1/Tcf7L2*-defficient cortices. Panels in Fig. 4h-k “depict double labelling of Sox2^+^ RGC and Tbr2^+^ IPC progenitors in single and double Tcf mutants. The number of Sox2^+^ progenitors decreased from 45.6 +/- 0.9% in controls to 19.5 +/- 0.5% in *Tcf7L2* and to 23.7 +/- 6.6% in *Tcf7L1/Tcf7L2* mutants while *Tcf7L1* single mutants displayed an unchanged number of Sox2^+^ cells in the VZ (44.4 +/- 0.9, *p* = 0.2) (Fig. 4f). Similarly, we observed a dramatic decrease of Tbr2^+^ cells in the SVZ in *Tcf7L2* and *Tcf7L1/Tcf7L2* mutants from 22.6 +/- 3.6% to 14.6 +/- 3.1% and 14.0 +/- 1.3%, respectively (Fig. 4h“-k“). Calculations from three independent experiments are shown in Fig. 4f-g. Altogether, lack of Tcf72 in the neocortical VZ leads to downregulation of canonical Wnt signalling and depletion of RGC which results in reduced IPC and hypoplasia of the neocortex.

## Discussion

**Fig. 5:**
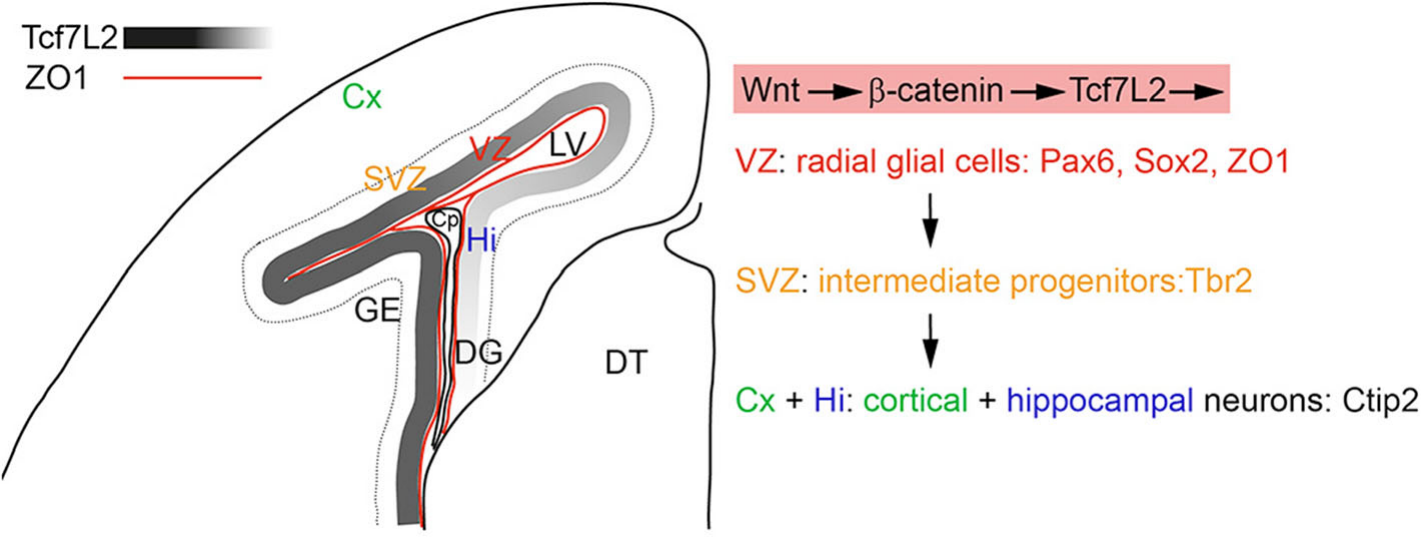
Summary diagram illustrating a part of the anatomical structure of one hemisphere of the embryonic forebrain at E17. Expression gradient of Tcf7L2 in the VZ and adherens junctions lining the lateral ventricle (red line) are shown. Dashed line depicts outer boundary of SVZ. Canonical Wnt signalling through Tcf7L2 affects cell identity of radial glial cells in the VZ and their proliferation. Applied cellular markers of neuronal progenitors for distinct stages of neuronal differentiation in the cortex are listed. Cp: choroid plexus, Cx: cortex, Hi: hippocampus, DG: dentate gyrus, DT: dorsal thalamus, GE: ganglionic eminences, LV: lateral ventricle, SVZ: subventricular zone, VZ: ventricular zone

In this work, we identified Tcf7L2 as the principal transcription factor mediating canonical Wnt signalling in the embryonic neocortex (summarized in Fig. 5). Although *Tcf7L2* is massively expressed in the dorsal thalamus its relatively weaker expression in the neocortical ventricular zone during neurogenic stages is sufficient to control transcription of Wnt target genes. Tcf7L2 has been shown to either activate or repress expression depending on the cellular context that may include the presence of other Tcf factors in the same cells, splicing variants etc. [14, 25–28]. Our experiments using BAT-Gal reporter mouse strain show that Tcf7L2 positively regulates Wnt target genes in the neocortical VZ. On the other hand, our data show that Tcf7L1 has negligible effect on neurogenesis in the mouse cortex although its expression in the VZ appears even stronger than that of Tcf7L2 (Fig. 1b-c). Using D6-Cre, which initiates recombination after E11.5 in the dorsal forebrain [22], deletion of Tcf7L1 alone had no effect on neurogenenis in our hands. Thus functions of Tcf7L1 and Tcf7L2 are not redundant in this area which may be explained by the fact that Tcf7L1 in most cases represses transcription [29]. This implicates that repression of Tcf targets is not necessary to set a proper transcriptional output of Wnt signalling in the cortex and that Wnt expression in the medial and lateral wall is sufficient to pattern arealization and control neurogenesis in the embryonic dorsal forebrain. The hippocampal defect in D6-Cre/Tcf7L1^+/+^/Tcf7L2^fl/fl^ resembles severed hippocampus in Lef1^−/−^ mice [16, 17] indicating that hippocampus development in the medial cortical wall requires both Lef1 and Tcf7L2. Tcf7L2^−/−^/Tcf1^−/−^ embryos die around 14.5 with multiple defects but the forebrain morphology was not studied in detail [30] and we thus present here the first study of Tcf7L2 function in this tissue.

Kuwahara and coauthors [31] reported that inhibition of Tcf7L1 by shRNA in vitro in dissociated cortical progenitors led to increased neuronal differentiation documented by increased levels of Tbr2 and β-tubulin. Further, experiments using overexpression or inhibition of Tcf7L1 *by* in utero electroporation of mouse cortices also concluded that Tcf7L1 is involved in maintaining RGC population by inhibiting neuronal differentiation [32]. Our genetic approach, on the other hand, did not confirm that Tcf7L1 plays a major role in cortical neurogenesis. This discrepancy could be explained that neural progenitors cultured in vitro are exposed to nonphysiological levels of Wnts in which repressor activity of overexpressed Tcf7L1 may influence neuronal differentiation.

D6-Cre/Tcf7L1/Tcf7L2 mutants display severed neuroepithelium (Figs. 2f, 3k‘). β-catenin associtates with N-cadherin and other proteins which participate in forming a polarized cytoarchitecture. Apically localized adherens junctions in radial glial cells are typical of the neuropithelium lining ventricles. Loss β-catenin or its constitutive activation leads to disruption of adherens junction and neuroepithelial integrity [8, 20]. Thus, Wnt loss- and gain-of function studies employing genetic ablation or modification of β-catenin yield complex phenotypic changes that are always accompanied with impaired adherens junction and cell-cell contacts. These defects include the loss of the neuroepithelial identity, the loss of RGC anchor in the VZ, rossette-like structures in the VZ or neuronal heterotopias [9]. In D6-Cre/Tcf7L1/Tcf7L2 mutants, however, β-catenin stability and intracellular localization is controlled by physiologically normal levels of Wnt signalling which should not cause aberrant cell contacts. Surprisingly, we detected a complete loss of adherens junctions as a consequence of Tcf7L2 genetic deletion. This suggests that Wnt-controlled gene expression, but not direct modification of β-catenin, is critical for adherens junctions formation. It remains to be determined what adherens junction components or determinants of VZ progenitors are direct or indirect Wnt targets. It is possible that Sox2, which is decreased in D6-Cre/Tcf7L2 mutant VZ, is directly controlled by Tcf7L2 as shown by binding of Tcf factors close to transcription start site of Sox2 [33].

Draganova et al. [13] used a specific conditional allele of β-catenin, Catnnb1^dm/flox^, for inactivation of canonical Wnt signalling in the cortex by Emx1-Cre. This Catnnb1^dm/flox^ variant maintained intact adherens junctions and yielded a decrease in RGC proliferation, subsequent depletion of RGC and IPC progenitor pool leading to reduced neuron number which corresponds to our data. Nevertheless, Draganova and coauthors did not detect disruption of the VZ neuroepithelium in Emx1-Cre/Catnnb1^dm/flox^ mutants up to E18.5, the latest analyzed stage. We report here that reduction of RGC population in D6-Cre/Tcf7L2 mutants precedes adherens junctions defect that becomes clearly visible at P0.

Although we observed efficient deletion of Tcf7L1 and Tcf7L2 at E15, the decreased number of RGC became apparent not before E17. A similar‚ delayed‘effect of Emx1-Cre/Catnnb1^dm/flox^ deletion on cortical neurogenesis was reported by Draganova et al. [13]. We show that IPC population was clearly reduced at P0, probably as a secondary effect of RGC depletion. This indicates that the temporal control of Tcf7L2 target genes regulating cell cycle and identity of RGC is not absolutely dependent on active Wnt signalling.

## Material and methods

### Mice

Generation and description of these mouse strains has been described previously: D6-Cre [22], Tcf7L1^fl/fl^ [23], ROSA26 [34]. Tcf7L2^fl/fl^ mouse strain was generated from Tcf7l2^tm1a(EUCOMM)Wtsi^ mice that were purchased from the European Conditional Mouse Mutagenesis Program EUCOMM (Welcome Trust Sanger Institute).

### Immunohistochemistry and in situ hybridization

Immunohistochemistry was performed on paraffin-embedded 5-μM thick sections using standard protocols. Primary antibodies: Lef1 (Cell Signaling 2230), Tcf7L1 (Santa Cruz, sc-8635), Tcf7L2 (Cell Signaling, 2569), Tcf7 (Tcf1, Cell Signaling 2203) Sox2 (Santa Cruz, sc-17320), β-galactosidase (Abcam ab9361, ab616), Tbr2 (Abcam, ab23345), ZO-1 (Santa Cruz, sc-10804), PCNA (Sigma, P8825), PH3 (Millipore, 06–570), Tuj1 (R&D, MAB1195). Secondary antibodies: biotinylated anti-rabbit or anti-mouse, Vectastain ABC Elite kit and ImmPACT DAB susbstrate (all Vector Laboratories). Donkey anti-rabbit, anti-mouse Alexa-Fluor 488 or 594 (Thermofisher).

In situ hybridization protocol and preparation of antisense β-galactosidase riboprobe was described in [20]. Confocal images were obtained on Leica SP5 confocal microscope. Wide-field fluorescence images were taken on Zeiss Imager.Z2.

## Additional file

Additional file 1: **Figure S1.** Radial glial cells and cortical neurons are reduced in their number in D6-Cre/Tcf7L1^fl/fl^/Tcf7L2^fl/fl^ mutants at E17. a-b Hematoxylin-eosin staining of coronal sections from controls and Tcf7L1/Tcf7L2 double mutants. c-d Tcf7L1 immunohistochemistry showing efficient deletion in the area of D6-Cre recombination. e-f Tcf7L2 immunohistochemistry showing efficient deletion in the cortical VZ. g-h“ Pax6 and Ctip2 double immunofluorescence illustrating a downregulation of RGC marker Pax6 and neuronal marker Ctip2 at E17 in Tcf7L1/Tcf7L2 double mutants. i-j“Sox2 and Tbr2 double immunofluorescence showing a negligible change in Sox2 expression in RGC and Tbr2 expression in intermediate neuronal progenitors at E17. (JPG 5316 kb)

Additional file 2: **Figure S2.** Radial glial cells, cortical neurons and structure are not altered in in D6-Cre/Tcf7L1^fl/fl^/Tcf7L2^fl/fl^ mutants at E15. a-c Hematoxylin-eosin staining of coronal sections from controls, Tcf7L2 single and Tcf7L1/Tcf7L2 double mutants. d-f Pax6 and Ctip2 double immunofluorescence with DAPI showing RGC marker Pax6. d‘-f‘Ctip2 immunofluorescence showing neuronal marker Ctip2 in the cortical plate (CP) at E15 in Tcf7L1/Tcf7L2 double mutants. g-i Tuj1 and Tbr2 double immunofluorescence counterstained with DAPI showing the cortical plate and intermediate neuronal progenitors in the SVZ at E15. g‘-i‘a higher magnification of Tbr2+ cells in the SVZ. . j-l PH3 and ZO1 double immunofluorescence with DAPI showing normal adherens junctions and normally dividing PH3^+^ progenitors at E15. j‘-l‘PH3 and ZO1 double immunofluorescence without DAPI. (JPG 2237 kb)

## Abbreviations

Cp: Choroid plexus
CP: Cortical plate
Cx: Cortex
DG: Dentate gyrus
DT: Dorsal thalamus
GE: Ganglionic eminences
Hi: Hippocampus
IPC: Intermediate progenitor cells
LV: Lateral ventricle
RGC: Radial glial cells
SVZ: Subventricular zone
VZ: Ventricular zone
