# Supplementary figures and images for: Tcf7L2 is essential for neurogenesis in the developing mouse neocortex
Source: Neural Dev. 2018 May 11;13:8. doi: 10.1186/s13064-018-0107-8 (PMC5946422; doi:10.1186/s13064-018-0107-8)

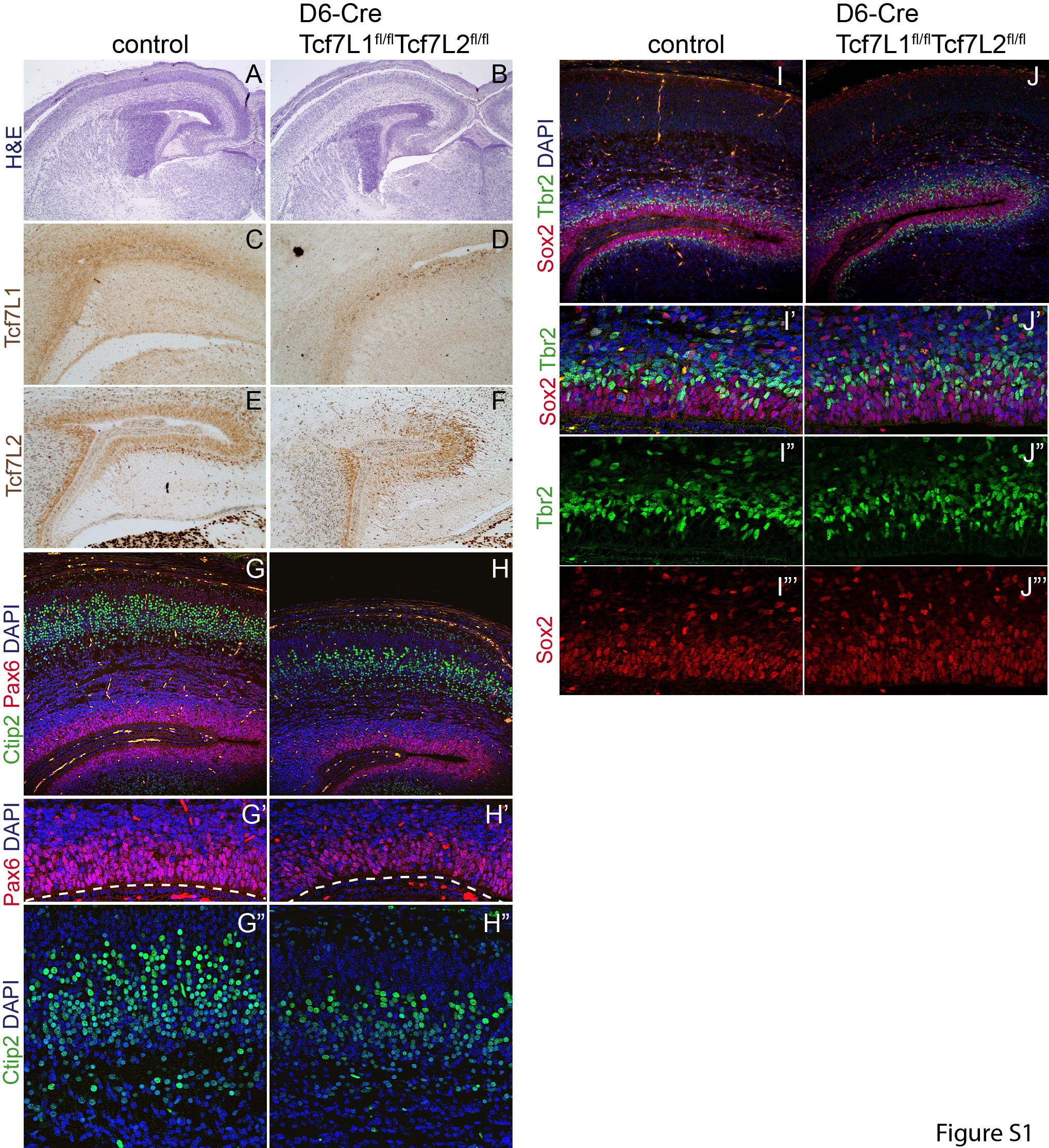

Supplement: Supplementary file 1 — Figure S1. Radial glial cells and cortical neurons are reduced in their number in D6-Cre/Tcf7L1fl/fl/Tcf7L2fl/fl mutants at E17. a-b Hematoxylin-eosin staining of coronal sections from controls and Tcf7L1/Tcf7L2 double mutants. c-d Tcf7L1 immunohistochemistry showing efficient deletion in the area of D6-Cre recombination. e-f Tcf7L2 immunohistochemistry showing efficient deletion in the cortical VZ. g-h“ Pax6 and Ctip2 double immunofluorescence illustrating a downregulation of RGC marker Pax6 and neuronal marker Ctip2 at E17 in Tcf7L1/Tcf7L2 double mutants. i-j“Sox2 and Tbr2 double immunofluorescence showing a negligible change in Sox2 expression in RGC and Tbr2 expression in intermediate neuronal progenitors at E17. (JPG 5316 kb) [file 13064_2018_107_MOESM1_ESM.jpg]

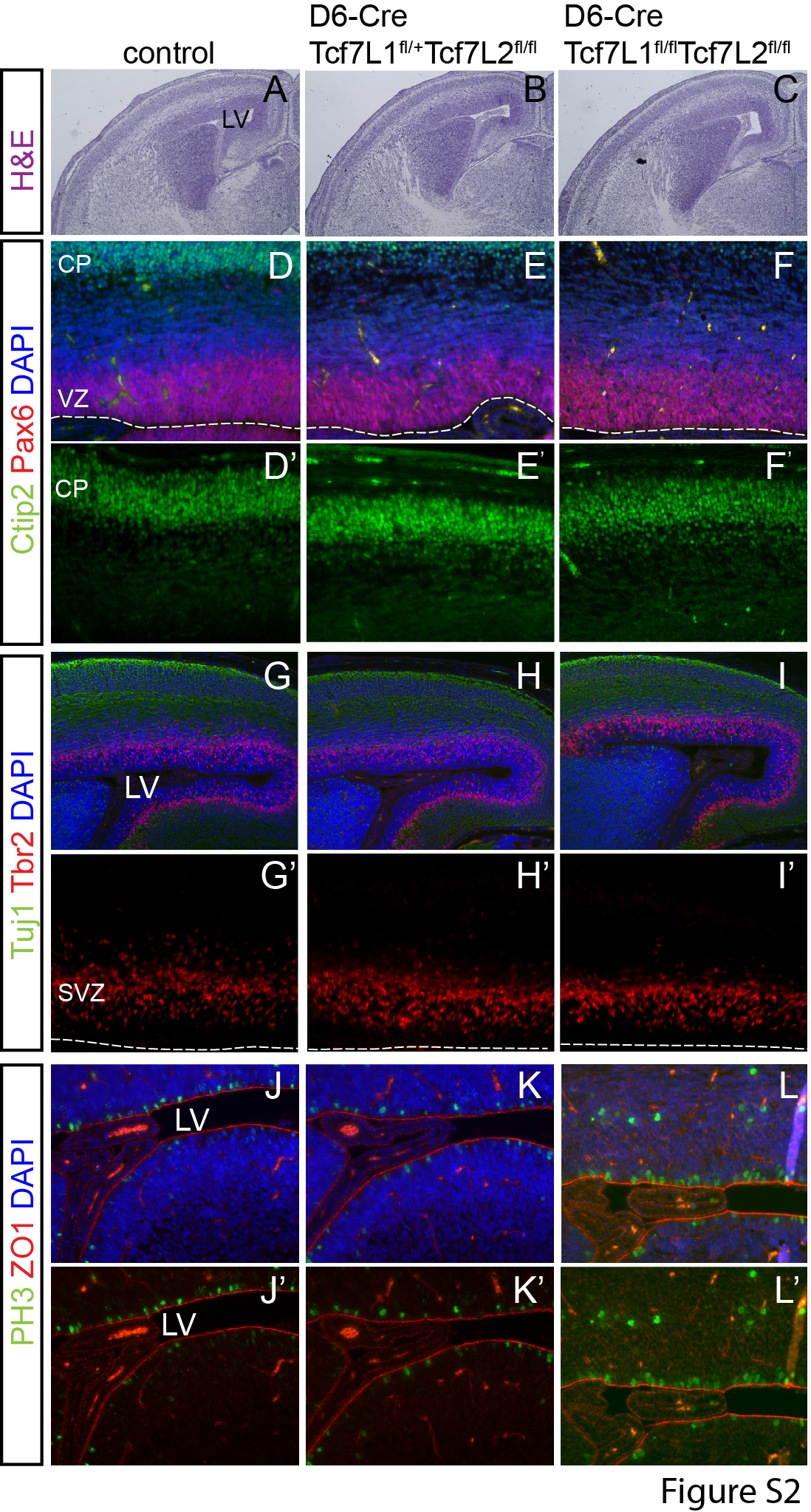

Supplement: Supplementary file 2 — Figure S2. Radial glial cells, cortical neurons and structure are not altered in in D6-Cre/Tcf7L1fl/fl/Tcf7L2fl/fl mutants at E15. a-c Hematoxylin-eosin staining of coronal sections from controls, Tcf7L2 single and Tcf7L1/Tcf7L2 double mutants. d-f Pax6 and Ctip2 double immunofluorescence with DAPI showing RGC marker Pax6. d‘-f‘Ctip2 immunofluorescence showing neuronal marker Ctip2 in the cortical plate (CP) at E15 in Tcf7L1/Tcf7L2 double mutants. g-i Tuj1 and Tbr2 double immunofluorescence counterstained with DAPI showing the cortical plate and intermediate neuronal progenitors in the SVZ at E15. g‘-i‘a higher magnification of Tbr2+ cells in the SVZ. . j-l PH3 and ZO1 double immunofluorescence with DAPI showing normal adherens junctions and normally dividing PH3+ progenitors at E15. j‘-l‘PH3 and ZO1 double immunofluorescence without DAPI. (JPG 2237 kb) [file 13064_2018_107_MOESM2_ESM.jpg]
